# Governance Roles and Capacities of Ministries of Health: A Multidimensional Framework

**DOI:** 10.34172/ijhpm.2020.39

**Published:** 2020-03-15

**Authors:** Kabir Sheikh, Veena Sriram, Benjamin Rouffy, Benjamin Lane, Agnes Soucat, Maryam Bigdeli

**Affiliations:** ^1^Alliance for Health Policy and Systems Research, World Health Organization, Geneva, Switzerland.; ^2^University of Chicago, Chicago, IL, USA.; ^3^World Health Organization, Geneva, Switzerland.; ^4^Health Systems Governance Collaborative, Geneva, Switzerland.

**Keywords:** Ministries of Health, Health Governance, Public Sector Capacity, Health Systems

## Abstract

The lack of capacity for governance of Ministries of Health (MoHs) is frequently advanced as an explanation for health systems failures in low- and middle-income countries (LMICs). But do we understand what governance capacities MoHs should have? Existing frameworks have not fully captured the dynamic and contextually determined role of MoHs, and there are few frameworks that specifically define capacities for governance. We propose a multidimensional framework of capacities for governance by MoHs that encompasses both the "hard" (*de jure* , explicit and functional) and "soft" (*de facto* , tacit, and relational) dimensions of governance, and reflects the diversification of their mandates in the context of the Sustainable Development Goals (SDGs). Four case studies illustrate different aspects of the framework. We hope that the framework will have multiple potential benefits including benchmarking MoH governance capacities, identifying and helping analyze capacity gaps, and guiding strategies to strengthen capacity.

## Introduction

Governance is said to refer to those processes that are formally or informally applied to distribute responsibility or accountability among actors in a given system.^1^ Ministries of Health (MoHs) occupy a unique role in the governance of health, given their exclusive constitutional or state-sanctioned mandates over the subject. MoHs provide direction and vision for the health system and are central to regulation and managing essential public health functions. At the country-level, MoHs can share and coordinate their governance roles with other organizational actors, such as councils, agencies and sub-national governments.

Inasmuch as the contexts of and demands on MoHs are evolving, their governance roles are also not static. The Sustainable Development Goals (SDGs), reflect the new and overdue imperative on ministries to exercise leadership beyond narrow sectoral boundaries.^2^ Major changes in the delivery, organization and management of health services, such as the rise of market-based health services and decentralization reforms, have created new exigencies for the stewardship, leadership and regulatory roles of MoHs.^3-5^ Today MoHs are expected to continue to manage public services and institutions, but also to proactively exercise leadership in pluralistic and multisectoral milieus, and also to predict and be prepared for emergent and future challenges.

The lack of capacity in MoHs for governance is frequently advanced as an explanation for health systems failures in low- and middle-income countries (LMICs).^6-9^ In 2017, the Health Systems Governance Collaborative – a community of diverse stakeholders including policy-makers, civil society actors and expert researchers with a secretariat in the World Health Organization (WHO) – determined that this should be a priority topic for its workplan. The Collaborative commissioned the development of this framework and an accompanying scoping review. Subsequently the Collaborative and the Alliance for Health Policy and Systems Research – a partnership hosted by WHO – jointly developed and finalized the framework following a round of reviews from potential users of the framework in MoHs and web-based consultations. The contribution of this paper is a coherent framework of the multiple dimensions that apply to MoH governance as it evolves: roles and capacities, mapped to performance areas. In doing so we draw on related existing frameworks of governance,^6,10-13^ and also more widely on the conceptual literature on health governance.^1,14,15^

## The Multiple Governance Roles of Ministries of Health

**Figure 1:**
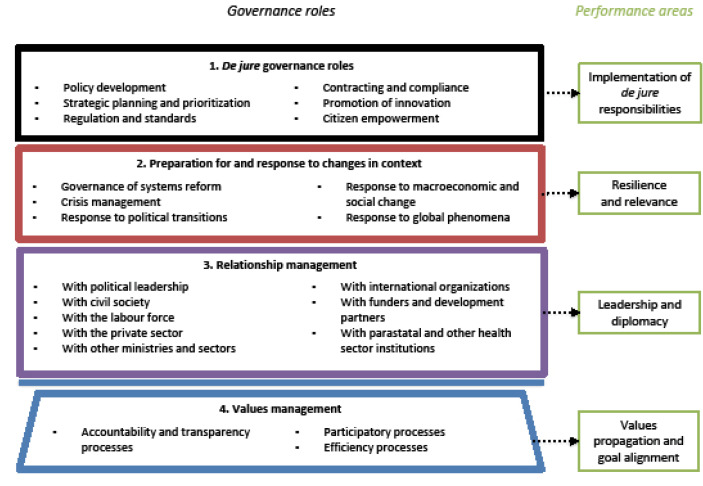
Governance Roles of MoHs. Abbreviation: MoHs, Ministries of Health.

Governance is a multidimensional process within which actors such as MoHs play specific roles. Drawing on diverse concepts and frameworks from the literature, including for stewardship functions,^13^ values and principles,^6,10-13^ contextual factors^13^ and outcomes or goals,^6,13^ we developed a framework for the governance roles of MoHs (Figure 1). We start by distinguishing between “de jure” governance roles, corresponding to explicit, formal or stated governance roles of MoHs, and other ‘soft’ or ‘tacit’ aspects of governance, which include MoHs’ roles in response to contextual changes, relationship management and values management, respectively. Here we define “role” as a function or part performed especially in a particular operation or process.^16^ Each role is linked to a key area of performance, where we define performance as the achievement of intended results, goals or objectives.^17^ Four types of governance roles of MoHs and their corresponding performance areas are outlined in Figure 1 and described below.

(1) “De jure” governance roles: MoHs have formal and sanctioned responsibilities over health, including a central role in health sector governance. While these responsibilities were traditionally described in terms of the management of public sector programmes and services, there has been a gradual expansion in these responsibilities in recent decades. Transformations in many LMICs, such as the growth of domestic private healthcare sectors, proliferation of donor agencies, and, more recently, a renewed focus on multisectoral action due to the SDGs, has expanded MoH’s remit in stewardship, regulation, resource management, intelligence management, citizen empowerment and the promotion of innovations.^2,4^ MoHs have the added pressure of these increasingly diverse tasks despite staffing limitations and frequent turnover, imbalances in staff skill mix, the demands of political leaders and international donors, and complex relationships with state and non-state actors at the national and subnational levels.^18,19^

(2) Preparation for and response to changes in context: Rapidly changing contexts – whether political, economic, ecological, or epidemiological – are a reality for MoHs. Yet, preparing for and responding to these changes represents an often unstated or tacit aspect of their governing responsibilities. MoHs are expected to navigate through mutable political climates, to prepare for and tackle health and environmental crises, adapt to economic and social change, and respond to global events and phenomena, among other changes.^3,19-21^

(3) Relationship management: MoHs actively manage an array of relationships, within and outside government. These relationships have been described as a spectrum, involving control, coordination, collaboration and communication, in different measure. Each relationship is also characterized by its own political and power dynamics. MoHs must negotiate with other sections of government, convene and collaborate with a host of non-state actors at the subnational, national and international levels, and manage regulatory relationships with stakeholders such as health professions or industries. Effective management of these relationships also ensures greater participation in policy-making, which in turn facilitates more ownership, diversity and effectiveness in the policy process.^22^ The SDGs have also underscored the need for MoHs to engage multiple sectors towards common goals.^23^

(4) Values management: Often least explicit is the role of MoHs in instituting and managing processes that uphold their governance roles, and that advance values and broader health and societal goals.^1^ Examples include processes to enhance accountability and transparency, increase efficiencies, and ensure participatory and inclusive decision-making.^22^ Values are often seen as latent, amorphous and shaped by political, social and organizational context, and are traditionally regarded to be difficult to change. However, distinct mechanisms to manage, enhance and promote these values can be put in place by MoHs.^10^

## Governance Capacities of Ministries of Health

All governance roles necessitate that MoHs possess specific capacities or abilities.^22^ Building capacity, particularly with regards to ‘soft’ governance roles such as relationship management or preparation for and response to changing context, is often unaddressed in guidance on strengthening the governance role of MoHs.^24,25^ Capacity, defined as “the ability to carry out stated objectives,”^26^ is a dynamic concept that refers to processes as well as outcomes.^27,28^

Capacity is required at multiple, intersecting levels in a health system for it to perform. Potter and Brough’s framework for health sector capacities^28^ identifies nine required categories for sequential strengthening. We draw on this framework adapting the six categories most relevant for MoH governance dimensions, namely:

- Structural capacity
- Mandated capacity (equivalent to Role Capacity in Potter and Brough)
- Personal capacity
- Workload capacity
- Performance capacity
- Supervisory capacity

**Figure 2:**
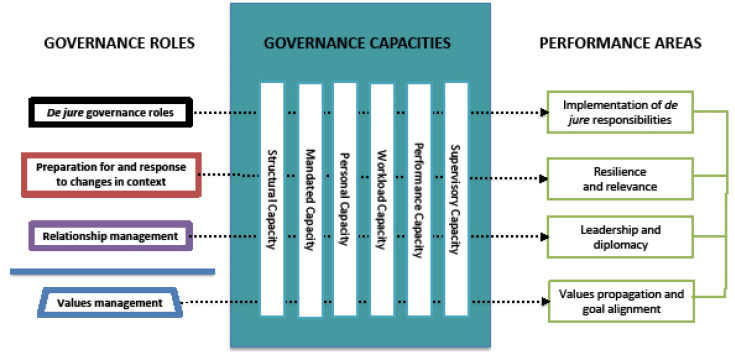
Multidimensional Framework for Governance Capacities of MoHs. Abbreviation: MoHs, Ministries of Health.

Table describes these six key types of capacities that are directly applicable to governance by MoHs, with examples for each type drawn from a scoping review on MoH governance capacities in LMICs.^29^ Figure 2 shows how governance roles and capacity categories map to performance areas.

**Table:** Types of Capacity for Governance by MoHs^a^

**Type of Capacity**	**Description**	**Examples**
Structural capacity	Refers to the existence of appropriate decision-making forums, systems of record keeping and mechanisms for accountability	National Health Assembly in Thailand enables engagement between civil society, government, academia and other sectors regarding health priorities^30^ Technical committees including Ministry of Health staff and health technology management professionals to monitor and approve health technology contracts in Benin^31^
Mandated capacity (or Role Capacity)	Refers to whether organizations have been given the appropriate authority or responsibility to make decisions and to undertake functions; may refer to formal and informal ownership of particular decision-making functions, and requires that an organization have sufficient power and legitimacy to undertake those functions	Law 7927 in Costa Rica enabled the legal authority of the Ministry of Health to act as steering entity for the health sector^21^ Establishment of the Tanzania Food and Drug Authority allowed for a comprehensive approach to regulating and licensing drug shops in Tanzania from 2002 onwards^32^
Personal capacity	Focuses on individual competencies, such as whether staff is sufficiently knowledgeable, skilled, motivated and competent to perform particular duties; skills may be technical, managerial, inter-personal, or other role-related skills	Process of Ministry of Health and Family Welfare staff directly managing SWAp in Bangladesh led to improvements in financial management^33^ Staff working in MoPH in Afghanistan underwent multi-year instructional programs, software and computer training, and individualized intensive training programs^34^ Ethiopia applied Business Process Reengineering to introduce results-based management to public sector institutions including Ministry of Health, which led to improvements in strategic planning, donor coordination, and managing donor funding^35^
Workload capacity	Focuses on quantitative aspects of capacity, such as sufficient numbers of staff, existence of clear, practical job description, appropriate skill mix, etc	Ministry of Health in Mexico developed technical capacity of staff to undertake analyses related to health-maximizing arguments (disease burden, cost-effectiveness) and non-health criteria (equity, implementation constraints) in order to strengthen Ministry’s approach to priority setting from 2003 onwards^36^
Performance capacity	Refers to the resources – tools, infrastructure, money, equipment and consumables – that are required to undertake these roles	Funding for operation of the Zambia Health Accreditation Council provided by donor funding^37^ Support from the MeTA facilitated establishment of Medicines Policy Unit within Ministry of Health in Kyrgyzstan^38^
Supervisory capacity	Concerns internal reporting and monitoring systems, accountability mechanisms, and availability of incentives and sanctions to facilitate the delivery of functions	Development of key performance indicators in Saudi Arabia to manage Ministry of Health’s initiative to broaden role of public private partnerships in the health sector^39^ Senegalese Ministry of Health adopted performance monitoring mechanisms for a school health program through qualitative stakeholder feedback, and through a quantitative analysis of school health facilities^40^

Abbreviations: SWAp, Sector-Wide Approach; MeTA, Medicines Transparency Alliance; MoHs, Ministries of Health; MoPH, Ministry of Public Health. ^a^Adapted from Potter and Brough.^28^

## Four Cases From Low- and Middle-Income Countries

The governance roles and the significance of different types of capacities are illustrated through the following case examples drawn from the literature from different LMICs. We have selected these four case examples from the scoping review, to illustrate the breadth of governance roles and diversity of approaches taken to strengthen capacity in different geographical contexts.

### (1) “De jure” Governance Roles: Strengthening the Capacity of the Afghanistan Ministry of Public Health to Regulate the for-Profit Private Health Sector^34,41^

In the past two decades, the Ministry of Public Health (MoPH) in Afghanistan has sought to reform its stewardship of the burgeoning for-profit private health sector. The Ministry began a project in 2008, supported by the United States Agency for International Development (USAID), the World Bank, and the EU, to strengthen its stewardship capacity. From 2004 onwards, a series of policies, strategies and regulations have been developed to strengthen the Ministry’s role in overseeing and managing this sector. From a *mandated capacity* standpoint, the 2008 National Development Strategy ‘appoints and mandates the MoPH as the steward of the health sector.’ The major piece of regulation that undergirds the mandated capacity for MoPH in this regard is the Private Health Centres Regulation that was formulated in 2012.

From a *structural capacity* standpoint, the Office of Private Sector Coordination was formed in 2009 to oversee functions related to for-profit providers; this Office was later elevated to a Directorate and formally made a part of the Ministry, including the sanctioning of civil service positions. The *performance capacity* of this Directorate is however still supported by donors. Other key structures include an Information and Communication Desk for private sector entities to engage with the Ministry, a quarterly Public-Private Dialogue forum chaired by the Minister, and the possibility of a Decision Review and Sanction Committee that will provide formal recourse for private providers.

*Personal capacity* amongst Ministry staff has been built through multi-year training programs, computer and software courses, and individualized intensive training through joint projects and collaboration. For example, USAID sponsored a detailed capacity assessment of the Ministry’s ability to implement the reform, and designed capacity building workshops on topics that ranged from the specific content of the legislation, to roles and responsibilities within the Ministry, to inspection practices, to leadership, to accounting and finance.

### (2) Response to Changes in Context: Strengthening the Mandated Capacity of the Ministry of Health in Costa Rica^21,42^

Following the economic crises of the 1980s, Costa Rica reportedly faced several challenges in this changing context, including bureaucratic inefficiencies, reduced services and public financing of health. By the 1990s, the government was actively considering avenues for reform. Contrary to privatization reforms that were gaining traction in other countries in the region, the government in Costa Rica focused on improving public health systems in the country through major structural changes, one of which was a renewed focus on the stewardship role of the Ministry of Health. A key focus of this effort was clarifying and strengthening the governance role of the Ministry in the health system.

In 1994, Law 7374 strengthened the *mandated capacity* of the Ministry of Health in stewardship, while transferring its existing health service delivery to another government agency, the Caja Costarricense de Seguro Social, enabling bureaucratic integration and more efficient health service delivery.^21^ The steering role of the Ministry was defined as “the political capacity to guide and lead the social production of health,” and the Ministry reorganized to achieve its functions in leadership and management, health surveillance, regulation and research and technological development. From a *personal capacity* standpoint, the Ministry created operating manuals for staff in new posts and invested in short- and long-term training of staff in these four new functions.^21^ For example, the Ministry funded the training of epidemiologists and health economists at the graduate-level.^21^ Other legal reforms were reportedly carried out in the 1990s and 2000s to further improve the mandated capacity of the Ministry including in the areas of regulation, surveillance and management.

###  (3) Relationship Management – the Observatório Network on Human Resources in Health in Brazil^43,44^

The Observatório Network on Human Resources in Health brings together the Ministry of Health, the Pan-American Health Organization (PAHO), university institutions, and research centers to engage with human resources for health issues in Brazil. This network has its roots in the 1970s and 1980s, when public health specialists began to come together on topics related to human resource for health, and health sector reform more broadly.^43^ Initially supported by PAHO, the Ministry of Health became an active partner in the late 1990s. In 1999, the Brazilian government legally recognized the network, giving its members the mandate to contribute to and inform the development of policy-relevant knowledge and exchange regarding human resources for health.^43,44^ The Ministry of Health serves as the secretariat for the network, with close support from PAHO; the proximity of these two institutions in Brasilia has further strengthened this partnership in supporting the network.^43^

*Structural capacity* driven by the legal foundation for the network from 1999 onwards ensured *performance capacity* in the form of funding from the Ministry of Health budget, facilitated by PAHO.^43,44^ The location of the network secretariat in the Ministry has also allowed for functions such as governance and financial management to become institutionalized. The network has also sought to maintain a balance between the independence of the network members and the needs of the government, which has been reported to be critical to its success.^43^

From a *workload capacity* standpoint, the secretariat within the Ministry is reportedly lean. Despite high turnover of staff within the Ministry, the role of PAHO has been helpful in ensuring continuity by dedicating one full-time staff member for human resources for health issues, and for the network.^43^ *Personal capacity* has been developed through shared experiences of many of the network members. Individuals sometimes transition between government, research and development partner employment, reportedly allowing for stronger consensus building due to their shared understanding of institutions in the network.^43^

### (4) Values Management: Healthcare Technology Management in the Public Health Sector in Benin^31^

The Ministry of Health in Benin, working with the Cooperation Benin-Union Européenne, initiated a phased approach to integrating a values orientation into the policy process for healthcare technology management. The approach also drew upon a participatory research effort that prioritized ‘the co-creation of knowledge between societal actors and experts.’^31^ In their assessment of the healthcare technology management system in Benin, the team reportedly found several challenges including capacity challenges related to the lack of policy and management tools, (such as an up-to-date list of essential medical devices and an annual maintenance budget), turnover in leadership of key departments, and limited opportunities for frontline staff (for example, engineers and technicians) to share their understanding of problems and solutions with more powerful decision-makers, and lack of accountability in the procurement process.^31^

Following these assessments, stakeholders developed a series of interventions and solutions to tackle these issues, undergirded by the goal of changing ‘values and practices of actors,’ and to promote new principles such as patriotism, civism, and depoliticized decision-making. Reportedly, interventions included mutual learning across stakeholders – including policy-makers and implementers – to develop a shared vision for reform, *personal capacity* initiatives such as the training of staff in new policy and management tools, *structural capacity* reform through the creation of a separate directorate and a budget line for healthcare equipment and maintenance, and the strengthening of *supervisory capacity* by developing a permanent technical committee for monitoring and evaluation.^31^ These interventions to improve healthcare technology management are efforts to put into practice key principles such as transparency, accountability, participation and efficiency in health system governance.^31^

## Conclusion

The framework presented and instantiated through case examples in this paper addresses a key gap in the knowledge by unpacking the hitherto “black box” of governance successes and failures attributed to MoHs. It differentiates the dimensions of governance and identifies within them a simple breakdown of categories of roles, capacities, and performance areas, linked by the governance functions MoHs need to perform in evolving contexts. The case examples highlight the interdependencies between different capacities. MoHs operate in complex contexts dominated by political aims and the interests of diverse actors. The framework also acknowledges this reality by including governance roles that are essentially political - namely relationship management and values management - in addition to the usually more formally mandated roles. Defining capacities for these hitherto “softer” governance roles is necessary to begin to address them transparently and coherently.^45^ It is worth noting that there may be instances where organizational goals for MoHs may not align with broader political aims, however this is outside the scope of this article.

Potential uses of the framework include providing the basis for mapping and gap analysis for MoHs and the identification of specific actions to strengthen governance capacity, for which it will be important to use it to develop customized assessment tools and field test them. Benchmarking is another key application of such field tested tools, especially for capacities that can be quantified or standardized, such as per capita workload capacity. The framework can also be used to conduct in-depth analyses of MoHs governance capacities, and as the basis for comparative analysis of governance roles and capacities across different MoHs and to promote learning between them.

Ultimately, we hope that this framework will drive further empirical study, identification of capacity needs, and short- and long-term capacity building efforts as well as more ambitious reforms of MoHs in view of strengthening health systems and governance. We encourage testing and refinement of the framework, with the belief that this will contribute to efforts to strengthen country MoH capacities for governance, in their multiple dimensions.

## Acknowledgements

We are grateful to Godelieve van Heteren, Sandy Campbell, and other members of the Health Systems Governance Collaborative for their guidance and feedback. We also thank the reviewers who provided thoughtful feedback at multiple stages of this project. This work was supported through funds from the WHO.

## Ethical issues

Not applicable.

## Competing interests

Authors declare that they have no competing interests.

## Authors’ contributions

KS and VS drafted the manuscript, received inputs from all authors and finalised the manuscript based on the inputs. All other authors contributed relevant inputs to the draft. All authors approved the final draft of the manuscript.

## Authors’ affiliations

^1^Alliance for Health Policy and Systems Research, World Health Organization, Geneva, Switzerland. ^2^University of Chicago, Chicago, IL, USA. ^3^World Health Organization, Geneva, Switzerland. ^4^Health Systems Governance Collaborative, Geneva, Switzerland.
